# M1 Macrophage-Derived Nanovesicles Repolarize M2 Macrophages for Inhibiting the Development of Endometriosis

**DOI:** 10.3389/fimmu.2021.707784

**Published:** 2021-07-20

**Authors:** Qiuju Li, Ming Yuan, Xue Jiao, Yufei Huang, Jing Li, Dong Li, Miaomiao Ji, Guoyun Wang

**Affiliations:** ^1^ Department of Obstetrics and Gynecology, Qilu Hospital of Shandong University, Jinan, China; ^2^ Cryomedicine Laboratory, Qilu Hospital of Shandong University, Jinan, China

**Keywords:** endometriosis, macrophage polarization, reprogramming, extracellular vesicles, treatment

## Abstract

**Background:**

Endometriosis is a common nonmalignant gynecological disorder that affects 10–15% women of reproductive age and causes several symptoms that result in decreased quality of life and a huge social burden. In recent decades, extracellular vesicles (EVs) have gained attention as a potential therapeutic tool; however, the therapeutic effects of EVs against endometriosis have not been reported. Accordingly, in this study, we investigated the feasibility of nanovesicles (NVs) derived from M1 macrophages (M1NVs) in treating endometriosis.

**Methods:**

M1NVs were prepared by serial extrusion. Co-culture assays were performed to investigate changes in tube formation and migration/invasion of eutopic endometrial stroma cells (ESCs) obtained from patients with endometriosis (EM-ESCs). A mouse model of endometriosis was established, and mice were treated with phosphate-buffered saline, M0NVs, or M1NVs to evaluate the efficacy and safety of M1NV for treating endometriosis.

**Results:**

M1NVs directly or indirectly inhibited the migration and invasion of EM-ESCs and reduced tube formation. In the mouse model, M1NVs suppressed the development of endometriosis through reprogramming of M2 macrophages, without causing damage to the organs.

**Conclusions:**

M1NVs inhibit the development of endometriosis directly, or through repolarizing macrophages from M2 to M1 phenotype. Hence, administration of M1NVs may represent a novel method for the treatment of endometriosis.

## Introduction

Endometriosis is a common nonmalignant gynecological disorder that affects 10–15% of women at reproductive age (1, 2). Endometriosis causes chronic pelvic pain, infertility, and progressive dysmenorrhea, leading to profound physical and psychological distress, thus disrupting the patient’s quality of life (3).

Although several therapeutic strategies have been established for the treatment of endometriosis, there is a high recurrence rate after drug and surgical treatments, thereby complicating therapeutic approaches (2).

It has been reported that the dysfunction of macrophages plays an important role in the development of endometriosis (4). Macrophages are usually divided into two phenotypes, M1 macrophages (classically activated macrophages) and M2 macrophages (alternatively activated macrophages). M1 macrophages secrete pro-inflammatory cytokines, including TNFα, IL-1, and IL-6, and release reactive oxygen species and reactive nitrogen species, which exert antiproliferative and cytotoxic activities. In contrast, M2 macrophages are considered anti-inflammatory cells, and they secrete IL-4, IL-10, IL-13, VEGF, and transforming growth factor β. M2 macrophages are associated with tissue repair, angiogenesis, neurogenesis, and protumor activity (5–8).

In patients with endometriosis, peritoneal macrophages are abnormally activated, which affects the clearance of cell debris and promotes cell proliferation and angiogenesis (4, 9, 10). Several studies have shown that peritoneal macrophages can be polarized to M2 macrophages in endometriosis mouse model or patients (11–13). An increased number of M2 macrophages in the peritoneal cavity promotes fibrosis and angiogenesis, further supporting the development of endometriosis. Bacci et al. and Haber et al. found that the depletion of the peritoneal macrophages using liposomes containing clodronate significantly blocks the development of endometriosis in mice (14, 15). Additionally, transplantation with M1 macrophages significantly reduces the growth of endometrial lesions, whereas transplantation with M2 macrophages promotes the development of lesions (15). Moreover, our previous research showed that exosomes derived from eutopic endometrial stroma cells (ESCs) of patients with endometriosis (EM-ESCs) alternatively activate macrophages (16). Thus, M2 macrophages have been suggested to promote the development of endometriosis, whereas M1 macrophages inhibit endometriosis. Hence, reprograming of M2 macrophages to M1 macrophages in the peritoneal cavity may be a potential strategy for treatment of patients with endometriosis. However, cell-related treatments are associated with various disadvantages, including poor survival of transplanted cells and difficulties with transportation and preservation.

Extracellular vesicles (EVs) and nanovesicles (NVs) have recently been shown to have potential applications as drug carriers and may be used to overcome the limitations of cell-related treatments (5, 6, 17–19). Previous studies have shown that M1 macrophage-derived nanovesicles (M1NVs) inhibit tumor development and enhance the anti-tumor effects of programmed cell death ligand 1 (5). Additionally, Kim et al. found that exosomes from M2 macrophages can facilitate the switching of M1 macrophages to M2 macrophages, and thus, further promote the wound healing (6). A recent study demonstrated that a two-component spike nanoparticle vaccine can potentially prevent of SARS-CoV-2 infection (18). EVs and NVs have also been shown to have applications in the treatment of tumors, trauma, or inflammation-related diseases. Although, several studies have demonstrated the critical roles of EVs in the development of endometriosis, few have elucidated the potential applications of EVs in the treatment of endometriosis (16, 20, 21).

Accordingly, in this study, we prepared M1NVs through serial extrusion and investigated whether these M1NVs could inhibit the migration and invasion of EM-ESCs and block angiogenesis. In addition, we explored whether M1NVs could reprogram M2 macrophages to the M1 phenotype and further suppress the development of endometriosis in a mouse model.

## Materials and Methods

### Human Sample Collection

Twenty-three patients with endometriosis and 10 patients with gynecological benign diseases other than endometriosis or adenomyoma, who underwent laparoscopic surgery at Qilu Hospital of Shandong University, were enrolled in this study. The diagnosis was confirmed with histopathology. All patients were 20–45 years of age and had a regular menstrual cycle. No hormone therapy or intrauterine contraceptive devices were used within 6 months prior to surgery. Endometrial samples were collected during surgery and immediately transferred to the laboratory on ice.

### Animals

Seven-week-old female C57BL/6 mice were obtained from Beijing Vital River Laboratory Animal Technology Co., Ltd (Beijing, China). All mice were maintained under specific pathogen-free conditions with a 12/12 h light/dark cycle, a controlled temperature of 25 ± 1°C and relative humidity of 55% (± 10%), and free access to food and water. Mice were allowed to acclimate for 10 days before treatment, during which the estrous cycle stages were monitored with vaginal smears. Only mice with normal estrous cycle, which were clear from the vaginal smears were used in experiments.

### Construction of an Endometriosis Model and Treatment of Mice

A mouse endometriosis model was constructed as previously described, with slight modifications (22). Briefly, donor and recipient mice were injected with estradiol benzoate at a dose of 3 μg/mouse (subcutaneous, s.c.; Aladdin, Shanghai, China) to synchronize the cycle 3 days before intraperitoneal injection of the uterus fragments. Donor mice were sacrificed, and uteri were removed. All uteri were mixed to eliminate differences among mice and then cut into fragments smaller than 1 mm^3^. After mixing with phosphate- buffered saline (PBS), fragments were divided into equal parts and then randomly injected into the peritoneal cavities of recipient mice. Three days after intraperitoneal injection, recipient mice were administered again with estradiol benzoate to enhance the development of endometriosis. Eighteen recipient mice injected with the uterus fragments from nine donor mice were randomly divided into three groups (one in group EM-M1NV died before the injection of M1NVs). Mice in the endometriosis (EM) group (n = 6), endometriosis plus M0NV treatment (EM-M0NV) group (n = 6), and endometriosis plus M1NV treatment (EM-M1NV) group (n = 5) were treated with equal volumes of PBS, M0NVs, and M1NVs, respectively, every 3 days from day 7 after intraperitoneal injection; the dose of NVs was 50 μg/mouse. Mice in the control group were injected with estradiol benzoate (s.c.) and PBS (i.p.) at the same dose and time point. All mice were sacrificed 14 days after injection of the uterus fragments.

### Primary Cell Isolation and Identification

Human ESCs were isolated from the endometrium, as previously described (23). The eutopic endometrium of patients with endometriosis or normal endometrium from patients with other gynecological benign disease, including ovarian benign epithelial tumours or mature teratomas, was collected during surgery. Samples were cut into pieces smaller than 1 mm^3^ after washing with PBS. Next, the pieces were digested in a collagenase mixture containing 0.25% (w/v) collagenase II and 0.125% (w/v) collagenase IV (Worthington, Biochemical Corp., Lakewood, NJ, USA) at 37°C until the mixture became transparent. The mixture was sequentially filtered through 100 and 40μm cell sieves, and ESCs were then collected from the filtered mixture, resuspended in Dulbecco’s Modified Eagle Medium/F12 (Gibco, Beijing, China) containing 10% (v/v) fetal bovine serum (FBS; Gibco), and cultured at 37°C in an atmosphere containing 5% CO_2_. Both immunofluorescence staining and flow cytometry (FCM) were used to identify the purity of ESCs by detecting the expression of the stromal marker vimentin.

Human umbilical vein endothelial cells (HUVECs) were isolated from normal human umbilical veins with collagenase. Approximately 10 mL of collagenase mixture, containing 0.25% (w/v) collagenase II and 0.125% (w/v) collagenase IV, was pumped into 15 cm of the umbilical vein, which was washed with PBS to remove the blood. HUVECs were collected from the digested mixture after digestion of the vein at 37°C for 45 min. HUVECs were resuspended in complete endothelial cell medium (Scien Cell) and identified by detection of CD31 expression using immunofluorescence staining and FCM.

### Immunofluorescence Staining

Primary ESCs, HUVECs, and *in vivo* polarized peritoneal macrophages were identified using immunofluorescence staining. Cells in 24 well plates were fixed with 4% paraformaldehyde for 20 min. After washing twice with PBS, cells were permeabilized with 0.25% Triton X-100 for 30min and washed twice with PBS. Next, the cells were blocked with 2% bovine serum albumin for 1 h at room temperature to avoid nonspecific binding of antibodies. Anti-human vimentin antibody (dilution: 1:400; Cat. no. ab92547; Abcam) was used to identify primary ESCs, and anti-human CD31 antibody (dilution: 1:500; Cat.no. ab28364; Abcam) was used to identified primary HUVECs. Anti-mouse F4/80 antibody (dilution: 1:500; Cat. no. GB11027; Servicebio, Wuhan, China) was used to identify macrophages before the test of polarization. Anti-mouse inducible nitric oxide synthase (iNOS) antibody (Cat. no. ab15323, Abcam) and anti-mouse CD206 antibody (Cat. No. 60143-1; Proteintech, Hubei, China) were premixed at final concentrations of 1:100 and 1:10000, respectively, before probing macrophages. Corresponding primary antibodies were added to the wells and incubated overnight in a humidified chamber at 4°C in the dark. Cells were washed with PBS, incubated with fluorescein-conjugated secondary antibodies at room temperature for 2 h in the dark, and then rewashed 3 times with PBS in the dark. The nuclei were stained with 4′,6-diamidino-2-phenylindole (DAPI, Cat. no. ab104139; Abcam) for 5 min and then visualized and imaged.

### Cell Culture

RAW264.7 mouse peritoneal macrophages and THP-1 human monocytes were provided by Chengjiang Gao (Department of Immunology, School of Medicine, Shandong University, Jinan, Shandong, China). RAW264.7 cells were cultured in complete Dulbecco’s Modified Eagle Medium (Gibco) containing 10% (v/v) FBS and 1% (v/v) penicillin/streptomycin (Gibco). THP-1 cells were cultured in complete Roswell Park Memorial Institute 1640 (Gibco) supplemented with 10% (v/v) FBS and 1% (v/v) penicillin/streptomycin. Phorbol 12-myristate 13-acetate (100 ng/mL; Sigma-Aldrich, St. Louis, MO, USA) was used to induce THP-1 cells to macrophages. Macrophage polarization was induced by 100 ng/mL lipopolysaccharide (LPS, Sigma-Aldrich) for 24 h to yield M1 macrophages or 20 ng/mL interleukin-4 (IL-4; R&D Systems, Minneapolis, MN, USA) for 24 h to yield M2 macrophages.

### Exosome Isolation

Exosomes were isolated from macrophage culture supernatants as previously described (23). Briefly, after the cells grew to 60% confluence, the medium was replaced with exosome-depleted medium (supplemented with exosome-depleted FBS obtained by centrifugation at 100,000 × g over night at 4°C) with or without LPS or IL-4. Supernatants were collected 24 h later and centrifuged sequentially at 500 × g for 10 min, 2,000 × g for 20 min, 10,000 × g for 30 min, and 100,000 × g for 70 min at 4°C to remove cells, debris, and micro-vesicles. Exosomes were resuspended with PBS, centrifuged at 100,000 × g for 70 min, re-resuspended with 100 μL PBS, and stored at -80°C.

### Preparation of NVs

NVs were prepared as reported previously (5). Briefly, macrophages treated with or without LPS or IL-4 were washed twice and resuspended in cold PBS. The suspension was then extruded 11 times with a syringe through 5, 1, 0.4, and 0.22 μm polycarbonate membrane filters (Millipore). All operations were performed on ice to avoid clumping of the macrophages and maintain the bioactivity of the NVs. The NVs were then centrifuged at 100,000 × g for 70 min and washed once. Protein concentrations of NVs and exosomes were tested with a BCA Protein Assay Kit (Beyotime, China) according to the manufacturer’s protocol. The contents of NVs were tested with a QuickEasyTM Cell Direct RT-qPCR Kit (CAT. No. DRT-01011, FOREGENE, China) according to the manufacturer's protocol.

### Transmission Electron Microscopy

Transmission electron microscopy was used to evaluate the morphologies of exosomes and NVs. Exosomes or NVs were adsorbed to a carbon-coated electron microscopy grid and fixed with 3% glutaraldehyde. After washing with distilled water, samples were stained with 4% uranyl acetate and 1% methyl cellulose aqueous solution thereafter. The JEOL 1200EX instrument was used to observe and capture images after naturally drying the sample grids for more than 30min.

### Nanoparticle Tracking Analysis (NTA)

NTA was performed with ZetaView PMX 110 (Particle Metrix, Meerbusch, Germany) to quantify the number of particles and measure the particle size. Exosomes or NVs from different experimental groups were detected. Samples in 100 μL PBS were diluted 1000–10,000 times to yield a final concentration of ~10^7^ particles/mL. Data were analyzed using ZetaView 8.04.02.

### NV cellular Uptake Assay

M2 macrophages, ESCs, and HUVECs were cultured for 24 h before addition of M1NVs. M1NVs were labeled with PKH67 (Sigma- Aldrich) according to the manufacturer’s protocol and the labeled M1NVs were then added to the culture medium. FCM was used to analyze the percent internalization at 0, 2, 4, 8, 12, and 24 h after the addition of M1NVs. Fluorescence microscopy was used to observe and capture images of the cells at 4 h after the addition of M1NVs.

### Evaluation of Toxicity of M1NVs *In Vitro*

M2 macrophages, ESCs, and HUVECs were cultured for 24 h. Next, different concentrations (0, 10, 20, 40, 80, and 160 μg/mL) of M1NVs were added, and cells were incubated for 24 h. The apoptosis rates of the cells were evaluated using the FITC Annexin V apoptosis kit (B&D System).

### Polarization of Macrophages *In Vitro* and Analysis by FCM/Reverse Transcription Quantitative Real-Time Polymerase Chain Reaction (RT-qPCR)

Macrophages were seeded in 6-well plates and cultured for 24 h to allow attachment to the plate surface. IL-4 was added to induce M2 polarization of macrophages for 24 h. M1NVs (20 μg/mL) were added to the plates containing M2 macrophages, and the LPS or IL-4, at the corresponding concentration, added to the other wells as the positive control; an equal volume of PBS was added to another well as the negative control. Cells were harvested 24 h later for FCM and RT-qPCR analysis.

The cells were incubated in fixation/permeabilization buffer (BD Biosciences, USA) for 25 min at 4°C in the dark before staining. After washing twice with 1× permeabilization/wash buffer (BD Biosciences), cells were incubated with phycoerythrin (PE)-conjugated anti-mouse iNOS antibody (Cat. no. 12-5920-82; eBioscience, USA) at a concentration of 0.2 μg/μL for 30 min at 4°C in the dark. After washing with 1× permeabilization/wash buffer, cells were resuspended in 200 μL PBS for FCM analysis.

Total RNA was extracted from cells using TRIzol reagent (Invitrogen Life Technologies, Carlsbad, CA, USA) according to the manufacturer’s protocol. RNA (1 μg) was reverse transcribed into cDNA (TOYOBO, FSQ-201, Japan), and RT-qPCR was performed for quantification of gene expression (TOYOBO, QPK-201, Japan). All the procedures were conducted following the manufacturer’s protocol. Expression of markers of M1 macrophages, including *CD86*, *IL6*, *IL1B*, and tumor necrosis factor-α (*TNFA)*, and markers of M2 macrophages, including *ARG1*, *CD163*, *CD206*, and *IL10*, was quantified. Vascular endothelial growth factor A (*VEGFA*) expression was also quantified to assess the effects on angiogenesis. The relative fold changes were normalized to endogenous *GAPDH* mRNA expression by using 2^-ΔΔCt^ method. Primer sequences for each gene analyzed by RT-qPCR are shown in **Table 1**.

**Table 1:** The primer sequences for each gene analyzed by qRT-PCR.

Gene	Primers	
	Forward (5’→3’)	Reverse (5’→3’)
CD86	CGACGTTTCCATCAGCTTGTC	CGCGTCTTGTCAGTTTCCAG
iNOS	CCTGCTTTGTGCGAAGTGTC	CCCAAACACCAAGCTCATGC
IL-6	TGAGGAGACTTGCCTGGTGAA	CAGCTCTGGCTTGTTCCTCAC
IL-1β	GACCACCACTACAGCAAGGG	AGGGAAAGAAGGTGCTCAGGT
TNF-α	GGGCAGGTCTACTTTGGGAT	AGGTTGAGGGTGTCTGAAGG
ARG-1	TGACGGACTGGACCCATCTT	GGCTTGTGATTACCCTCCCG
CD163	TCTCTTGGAGGAACAGACAAGG	CCTGCACTGGAATTAGCCCA
CD206	GATTGCAGGGGGCTTATGGG	CGGACATTTGGGTTCGGGAG
IL-10	CCAGACATCAAGGCGCATGT	GATGCCTTTCTCTTGGAGCTTATT
VEGFA	GCAGAATCATCACGAAGTGGT	CCAGGGTCTCGATTGGATGG
GAPDH	GCACCGTCAAGGCTGAGAAC	TGGTGAAGACGCCAGTGGA

### Collection of Peritoneal Macrophages

Mice were sacrificed, and 1 mL cold PBS was injected into the peritoneal cavity and the peritoneal lavage fluid was collected after shaking for 30 s. Next, 10 mL (5 mL × 2) cold PBS supplemented with 2% FBS was injected into the peritoneal cavity and the peritoneal lavage fluid was harvested after shaking for 30 s. Approximately 500 μL of the peritoneal lavage fluid was centrifuge at 300 × g for 5 min and the cells were resuspended in Roswell Park Memorial Institute 1640 medium and seeded into each well of a 24-well plate to purify the macrophages for subsequent immunofluorescence staining. Four hours after seeding, the medium was changed, and after an additional 24 h incubation, the residual adherent cells were identified with FCM and immunofluorescence staining.

### Polarization of Peritoneal Macrophages *In Vivo*

Polarization of peritoneal macrophages *in vivo* was analyzed using immunofluorescence staining and FCM. Immunofluorescence staining was conducted as described in *Immunofluorescence Staining*.

For FCM, the remaining peritoneal lavage fluid was fixed with 2% paraformaldehyde on ice to avoid clumping of the macrophages. After blocking the FC receptors with anti-mouse CD16/32 antibodies, the pan-macrophage markers F4/80 (allophycocyanin conjugated; Cat. no. 17-4801; eBioscience) and CD11b (PerCP-Cyanine 5.5 conjugated; Cat. no. 45-0112; eBioscience) were added in a 100 μL cell suspension with approximately 10^5^ cells to final concentrations of 0.02 and 0.0025 μg/μL, respectively. Cells were incubated for 15 min at 4°C in the dark and washed with cold PBS twice. After centrifugation, cells were permeabilized in fixation/permeabilization buffer (BD Biosciences) for 25 min at 4°C in the dark and then washed with 1× permeabilization/wash buffer (BD Biosciences) twice. The cells were incubated with PE-conjugated anti-mouse CD206 antibody (Cat. no. 141705; Biolegend, USA) and PE-cyanine 7-conjugated anti-mouse iNOS antibody (Cat. no. 25-5920, eBioscience) for 30 min at 4°C in the dark at final concentrations of 0.2 and 0.0006 μg/μL, respectively. Cells were washed with 1× permeabilization/wash buffer and resuspended with PBS for FCM analysis.

Peritoneal macrophages positively expressed F4/80 and CD11b. Cells with high expression of both F4/80 and CD11b were considered large peritoneal macrophages (LPMs), whereas those with low expression of both F4/80 and CD11b were defined as small peritoneal macrophages (SPMs) (22). Combined with the expression of pan-macrophage markers, iNOS-positive and CD206-negative cells were identified as M1 macrophages, whereas CD206-positive and iNOS-negative cells were identified as M2 macrophages.

### Immunohistochemical and Hematoxylin and Eosin (H&E) Staining of Tissues

Four micron-thick paraffin-embedded tissue sections were dewaxed and rehydrated before H&E and immunohistochemical staining. Slides were stained with hematoxylin (Servicebio, China) for 4 min and eosin (Servicebio) for 5 min. For immunohistochemical staining, slides were blocked with 2% bovine serum albumin for 2 h, and a mixture of primary antibodies, including anti-E-cadherin (dilution: 1:200, Cat. no. AF748; R&D Systems) and anti-vimentin antibodies (dilution: 1:200, Cat. no. ab92547, Abcam), anti-F4/80 and anti-iNOS antibodies were used to immunostain the tissue sections. Slides were incubated in a humidified chamber overnight at 4°C in the dark. After washing twice with PBS, the slides were incubated with premixed fluorescein-conjugated secondary antibodies containing Dylight 488-conjugated donkey anti-rabbit (dilution: 1:400; Cat. no. ab96919; Abcam) and Dylight 594-conjugated donkey anti-goat secondary antibodies (dilution: 1:400; Cat. no. ab96933; Abcam) at room temperature for 2 h in the dark, and after washing with PBS, were mounted with DAPI (Cat. no. ab104139, Abcam). Images were captured using a fluorescence microscope (Olympus BX53; Olympus, Tokyo, Japan).

### ESC Migration and Invasion Assay

To investigate the impact of M1NVs on the migration and invasion capacities of EM-ESCs, we performed cell migration and invasion assays. Transwells (Corning, USA) with 8.0 µm Pore Polycarbonate Membrane Inserts were precoated with (invasion assay) or without (migration assay) 100 μL Matrigel Matrix (Corning) on ice and incubated at 37°C for 30 min before seeding the cells. To investigate the direct effects of M1NVs, ESCs were pre-incubated with 20 μg/mL NVs derived from different types of macrophages for 24 h. The ESCs were then seeded in the upper chambers, which contained 5% FBS, whereas the lower chambers contained 600 μL medium supplemented with 20% FBS. After 24 h of incubation, cells above the membrane were removed using a cotton swab. The remaining cells were fixed with 4% paraformaldehyde for 10 min, stained with 0.1% crystal violet, and photographed under a microscope (Olympus BX53; Olympus, Tokyo, Japan). To investigate the indirect effects of M1NVs, the macrophages were induced with LPS, IL-4, or M1NVs; ESCs were seeded into the upper chambers of the inserts, whereas different types of macrophages were seeded into the lower chambers. Medium containing 10% FBS was added to both the upper and lower chambers. Cells that migrated or invaded were counted using Image J v1.8.0 (National Institutes of Health, Bethesda, MD, USA).

### *In Vitro* Tube Formation Assay

Tube formation assays were conducted to investigate the effects of M1NVs on angiogenesis. HUVECs were pre-incubated with NVs from macrophages induced with LPS or IL-4, or were cocultured with macrophages induced by LPS, IL-4, or M1NVs for 24 h. To investigate whether reprogrammed M2 macrophages (Re-M2, M2 macrophages treated with M1NVs for 24 h) could alter the proangiogenic capacity of EM-ESCs, EM-ESCs were cocultured with M0, M1, M2, or Re-M2 macrophages for 24 h. Subsequently, HUVECs were co-cultured with control ESCs (Co-ESCs), EM-ESCs, or pretreated EM-ESCs for 24 h. Next, 96-well plates precooled at -20°C overnight were coated with 40 μL growth-factor-reduced Matrigel Matrix (Corning) at 37°C for 30 min, and 1 × 10^5^ HUVECs in 100 μL endothelial cell medium were seeded onto the plates. Tube formation was observed and imaged 4 h later, and quantification was performed using Image J v1.8.0 (National Institutes of Health).

### Measurement of Endometriosis Lesions

All endometriosis lesions were harvested after mice were sacrificed, and the number of lesions was recorded. The longest diameter “a” and shortest diameter “b” of each lesion were measured with vernier calipers. The lesion volumes were estimated according to an ellipsoidal calculation using the formula V = a × b^2^ × 0.5. In addition, the weights of the lesions were measured.

### Tracing NVs *In Vivo*

For investigating the distribution of NVs *in vivo*, NVs labeled with PKH67 were injected into the peritoneal cavity of endometriosis mouse model. Peritoneal lavage fluid was collected as description in *Collection of Peritoneal Macrophages*. PKH67, F4/80 and CD11b positive cells were considered as macrophages that had phagocytosed NVs. Organs, including the liver, heart, spleen, lungs, kidneys, adipose tissue near the bladder, and bladder, were harvested immediately after sacrificing mice. All organs and lesions were placed on a nonreflective black cardboard as background, and the fluorescence intensity was measured with the *in vivo* imaging system (IVIS) Spectrum instrument (PerkinElmer, USA).

### Statistical Analysis

GraphPad Prism 8.0.1 (GraphPad Software, Inc., La Jolla, CA, USA) was used for statistical analysis. All data were tested for normality with the Kolmogorov–Smirnov test. One-way analysis of variance was used to compare differences among groups, and results with *P* values less than 0.05 were considered significant. All data are presented as means ± standard deviations, and each experiment was repeated three times.

## Results

### Characteristics of NVs Derived From Macrophages

**Figure 1:**
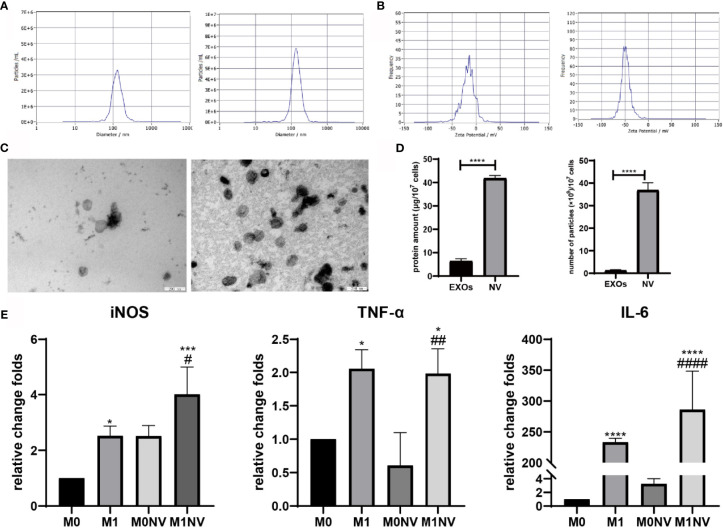
Characteristics of nanovesicles. **(A)** Diameter of exosomes (left) and NVs right). **(B)** Zeta potential of exosomes (left) and NVs (right). **(C)** Morphology of exosomes (left) and NVs (right). Scale bar =200nm. **(D)** Protein amount and particles of exosomes or NVs derived from10^7^ cells. **(E)** qRT-PCR revealed that M1NVs contained more mRNA of pro-inflammatory factors and M1 macrophage markers, indicating that M1NVs could effectively retain the functional characteristics of M1 cells. (*, ***, ****P < 0.05, P < 0.001. P < 0.0001 when compared to M0. ^#^, ^##^, ^####^P < 0.05, P < 0.01, P < 0.0001 when compared to M0NV).

NVs were obtained from RAW264.7 cells treated with or without LPS or IL-4 by serial extrusion through polycarbonate membranes. NTA of the NVs showed a peak diameter of 137 nm, similar to the diameters of exosomes. Electrophoretic light scattering showed a negative zeta potential for both NVs and exosomes. The amount of protein and number of particles in NVs were four and ten times higher, respectively, than those of exosomes derived from the same number of cells under natural conditions (**Figure 1**). These findings suggested that NVs produced by serial extrusion had physical characteristics similar to that of exosomes, but their production was higher than that of exosomes obtained from the same number of cells. Since M1NVs were prepared by serially extruding M1 macrophages, we evaluated the relative change folds of mRNA, including iNOS, TNF-α and IL-6, which are pro-inflammatory factors and M1 macrophage markers, in M1 and M1NVs *via* qRT-PCR. The results revealed that M1NVs contained more mRNA of pro-inflammatory factors and M1 macrophage markers (**Figure 1E**), indicating that M1NVs could effectively retain the functional characteristics of M1 cells.

### M1NVs Were Internalized by Primary Cells and Macrophages, and Reprogrammed M2 Macrophages Into M1 Macrophages

**Figure 2:**
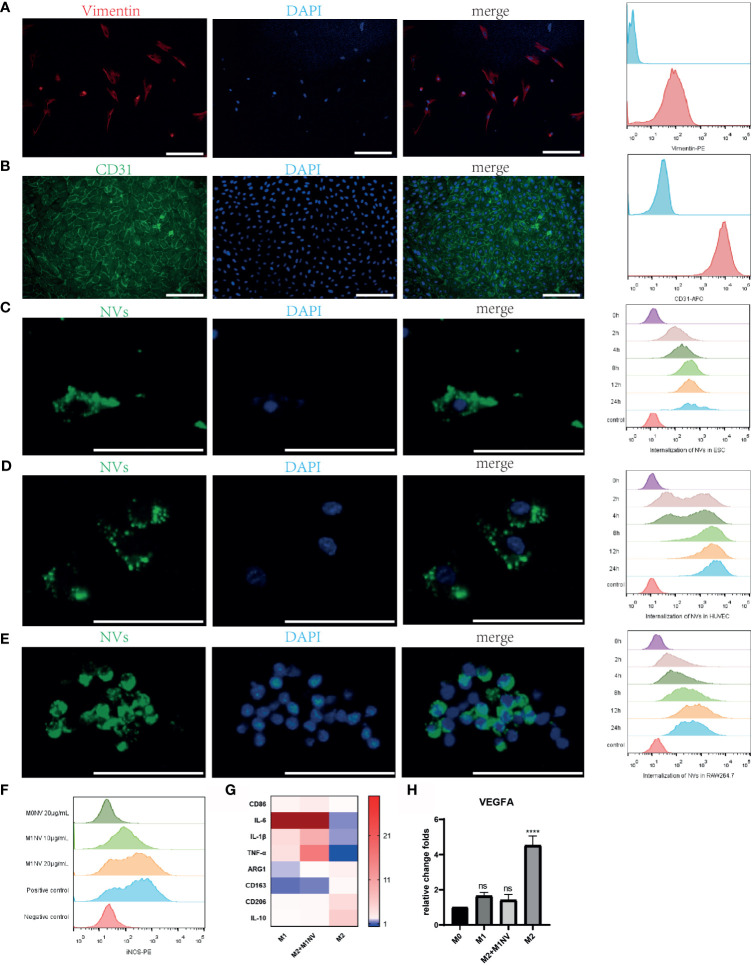
M1NVs were internalized by primary cells and macrophages and reprogrammed M2 into M1 macrophages. **(A)** ESCs derived from endometrium expressed stroma marker Vimentin (Scale bar = 100 μm). **(B)** HUVECs derived from normal human umbilical veins expressed CD31, the endothelial marker (Scale bar = 100 μm). **(C–E)** NVs labeled by PKH67 were internalized in the ESCs, HUVECs and macrophages. With the passage of time, the number of NVs internalized into the cells was increasing (Scale bar = 100 μm). **(F)** M2 macrophages were polarized into M1 by M1NVs, and 20μg/mL of M1NVs were sufficient to play the role. **(G)** RT-qPCR revealed the up-regulated M1 markers (*CD86*, *IL6*, *IL1B, and TNFA*) and down-regulated M2 markers (*ARG1*, *CD163*, *CD206*, *IL10*) in Re-M2 macrophages. **(H)** Re-M2 macrophages expressed lower level *VEGFA* than M2 macrophages. ns, no significance when compared to M0. ****P<0.0001 when compared to M0.

Primary ESCs and HUVECs were identified with immunofluorescence staining and FCM analysis with a purity of almost 100% (**Figures 2A, B**). PKH67-labeled NVs were internalized by ESCs, HUVECs, and RAW264.7 cells after coculture. Moreover, over time, the number of NVs internalized by the cells increased, as shown by FCM analysis (**Figures 2C–E**).

After treatment with different concentrations of M1NVs or M0NVs, M2 macrophages were analyzed by FCM. Compared with M0NVs, M1NVs could effectively reprogram M2 macrophages into M1 macrophages and 20 μg/mL M1NVs were sufficient for this repolarization (**Figure 2F**). RT-qPCR showed that Re-M2 macrophages expressed markers similar to those of M1 macrophages, including *CD86*, *IL-6*, *IL-1β*, and *TNF-α*, whereas the expression of the M2 macrophage markers, including *ARG1*, *CD163*, *CD206*, and *IL-10* was downregulated (**Figure 2G**). Moreover, M1NVs could downregulate *VEGFA* expression in Re-M2 macrophages (**Figure 2H**).

### M1NVs Inhibited the Migration and Invasion of EM-ESCs Directly or Through Reprogramming of M2 Macrophages

**Figure 3:**
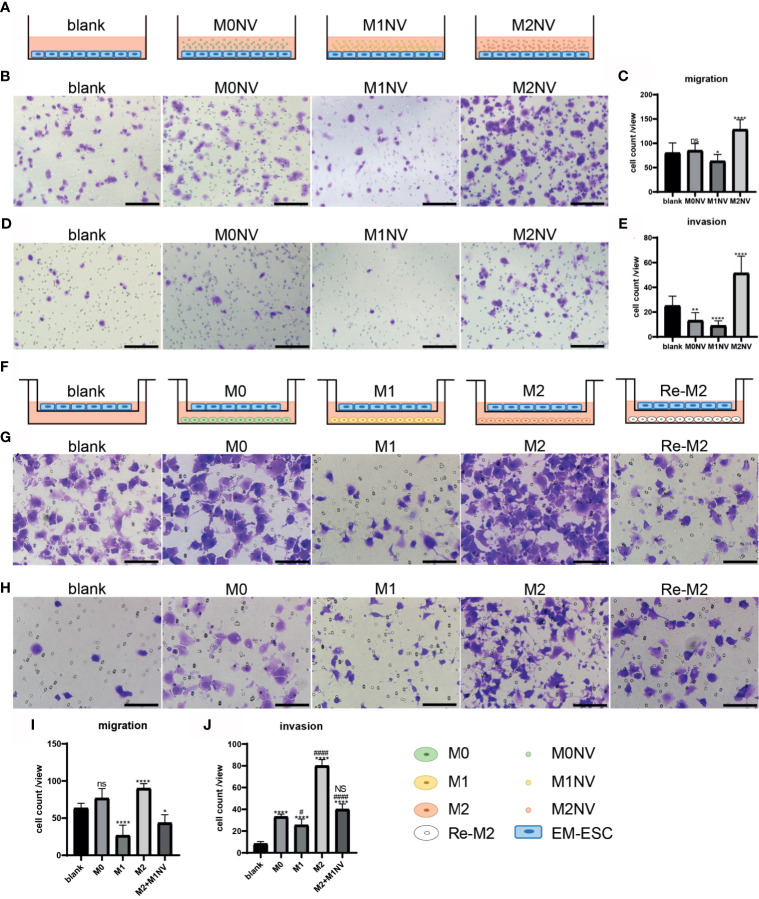
M1NVs could inhibit the migration and invasion of EM-ESCs directly or through reprogramming of M2 macrophages. **(A, F)** Co-culture pattern diagram of the migration and invasion assays. **(B–E)** Migration **(B, C)** and invasion **(D, E)** of EM-ESCs treated with NVs derived different type of macrophages, and M1NVs could inhibit the migration and invasion of EM-ESCs (Scale bar =100 μm). **(G–J)** Migration **(G, H)** and invasion **(I, J)** of EM-ESCs treated by different type of macrophages (Scale bar =100 μm). (ns: no significance when compared with blank. NS: no significance when compared with M0. *P<0.05; **P<0.01, ****P<0.0001 when compared with blank. ^#^P<0.05; ^####^P<0.0001 when compared with M0 macrophages.

When EM-ESCs were treated for 24 h with M1NVs alone as shown in **Figure 3A**, significantly fewer cells migrated from the upper to the lower chamber compared with that after incubation with M0NVs. Interestingly, M2NV treatment significantly promoted EM-ESC migration (**Figures 3B, C**). Similar results were observed in the invasion assay (**Figures 3D, E**).

Following co-culture with different types of macrophages, as shown in **Figure 3F**, the migration and invasion capacities of EM-ESCs were significantly inhibited by M1 macrophages. Although M2 macrophages promoted the migration and invasion of EM-ESCs, Re-M2 macrophages decreased the capacity of EM-ESCs to migrate and invade, similar to that observed with M1 macrophage treatment (**Figures 3G–J**).

### M1NVs Directly and Indirectly Inhibited Tube Formation *In Vitro*

**Figure 4:**
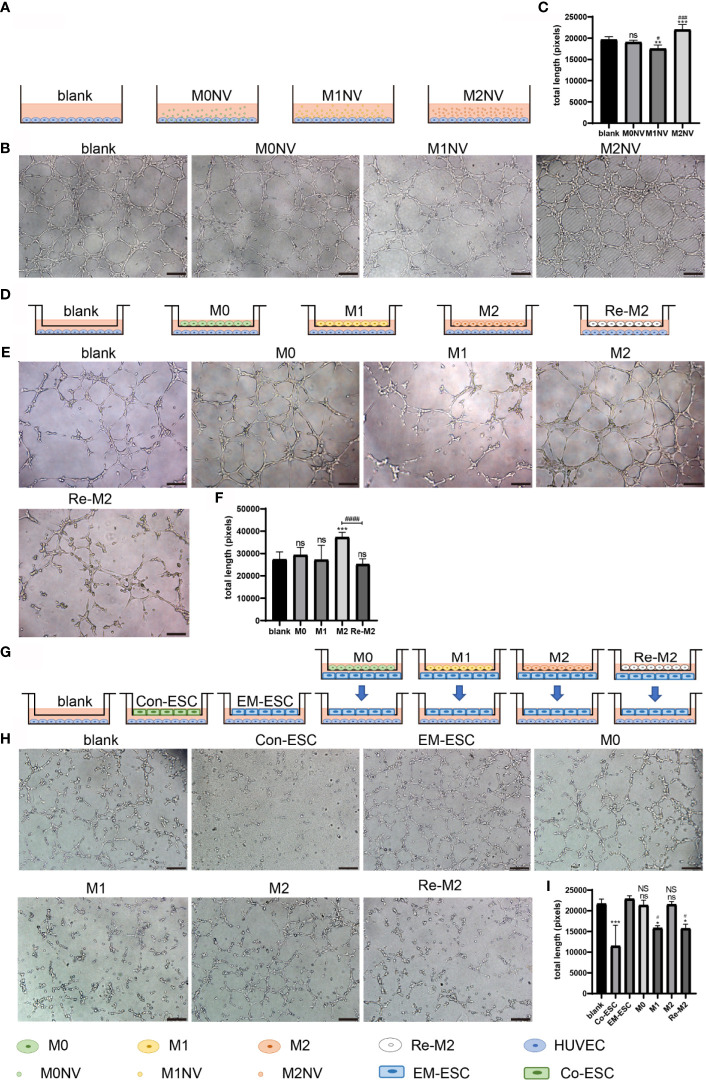
M1NVs could inhibit HUVEC tube formation in *vitro* in direct and indirect way. **(A, D, G)** Co-culture pattern diagram of HUVEC tube formation. **(B, C)** Tube formation of HUVECs treated with NVs directly (Scale bar =100 μm). (ns: no significance when compared with blank. **P<0.01, ***P<0.001 when compared with blank. ^#^P<0.05, ^###^P<0.001 when compared with M0NVs). **(E, F)** Tube formation of HUVECs treated with different types of macrophages (Scale bar =100 μm). (ns: no significance when compared with blank. ***P<0.001 when compared with blank. ^####^P<0.0001 when compared with M2). **(H, I)** Tube formation of HUVECs treated with ESCs which were cocultured with different types of macrophages (Scale bar = 100 μm). (*P<0.05, ***P<0.001 when compared with blank. ns, No significance when compared with Co-ESCs. NS, No significance when compared with EM-ESCs. ^#^P<0.05 when compared with EM-ESCs).

After treatment with different NVs, as shown in **Figure 4A**, or co-culturing with different types of macrophages, as shown in **Figure 4D**, HUVEC tube formation capacity was significantly altered. Specifically, M1NVs inhibited tube formation, whereas M2NVs promoted tube formation compared with M0NVs (*p* < 0.05; **Figures 4B, C**). Tube formation by HUVECs treated with Re-M2 macrophages was significantly reduced compared with that by cells treated with M2 macrophages and was similar to the tube formation by cells treated with M1 macrophages (**Figures 4E, F**).

In our previous study, we showed that exosomes from the endothelium of patients with endometriosis promoted tube formation *in vitro* (23). In this study, we further confirmed that EM-ESCs promoted tube formation *in vitro* compared with ESCs from patients without endometriosis (Co-ESCs). Interestingly, after co-culturing with M1 and Re-M2 macrophages, as shown in **Figure 4G**, the tube formation capacity of EM-ESCs was reversed (**Figures 4G–I**). Taken together, these results suggested that M1NVs inhibited tube formation directly or through reprogramming of M2 macrophages, which could alter tube formation induced by EM-ESCs.

### M1NVs Inhibited the Development of Endometriosis *In Vivo*

**Figure 5:**
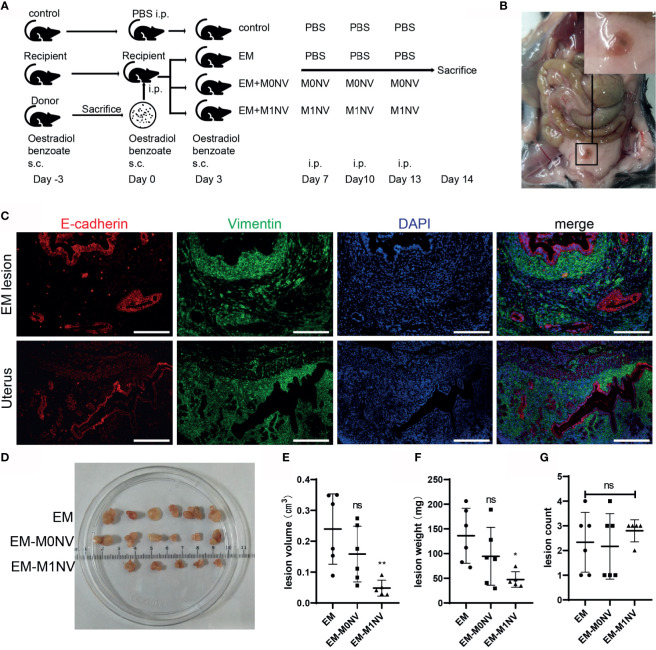
M1NVs inhibited the development of endometriosis in a murine model. **(A)** Flow diagram of the experiment *in vivo*. **(B)** Typical ectopic endometrial lesion could be found in the peritoneal cavity of the mouse model 14 days after uterus fragment injection. **(C)** Immunofluorescence staining showed the ectopic lesion had typical endometrial glands (E-cadherin positive, red) and stroma (Vimentin positive, green) (Scale bar =100 μm). **(D)** Ectopic lesions in different groups. **(E, F)** Treatment of M1NVs significantly reduced the volume and weight of lesions. (ns: no significance when compared with EM. *P<0.05, **P<0.01 when compared with EM). **(G)** The count of lesions was not different among groups.

Female C57BL/6 mice with regular estrous cycle cleared from vaginal smears were treated as shown in **Figure 5A**. Mice were sacrificed 14 days after injection of uterine fragments. All mice in the EM, EM-M0NV, and EM-M1NV groups showed successful induction of macroscopic endometriosis lesions, with adhesion to the surrounding organs (**Figure 5B**). Tissue immunofluorescence analysis showed classical endometrial glands (with high E-cadherin expression) and stroma (with high vimentin expression; **Figure 5C**).

Notably, M1NV treatment significantly reduced the lesion volume and weight compared with M0NV and PBS treatment (**Figures 5D–F**), while did not reduce the number of lesions (**Figure 5G**).

### M1NVs Inhibited the Development of Endometriosis *In Vivo* Through Programming of Peritoneal Macrophages Into M1-Phenotype

**Figure 6:**
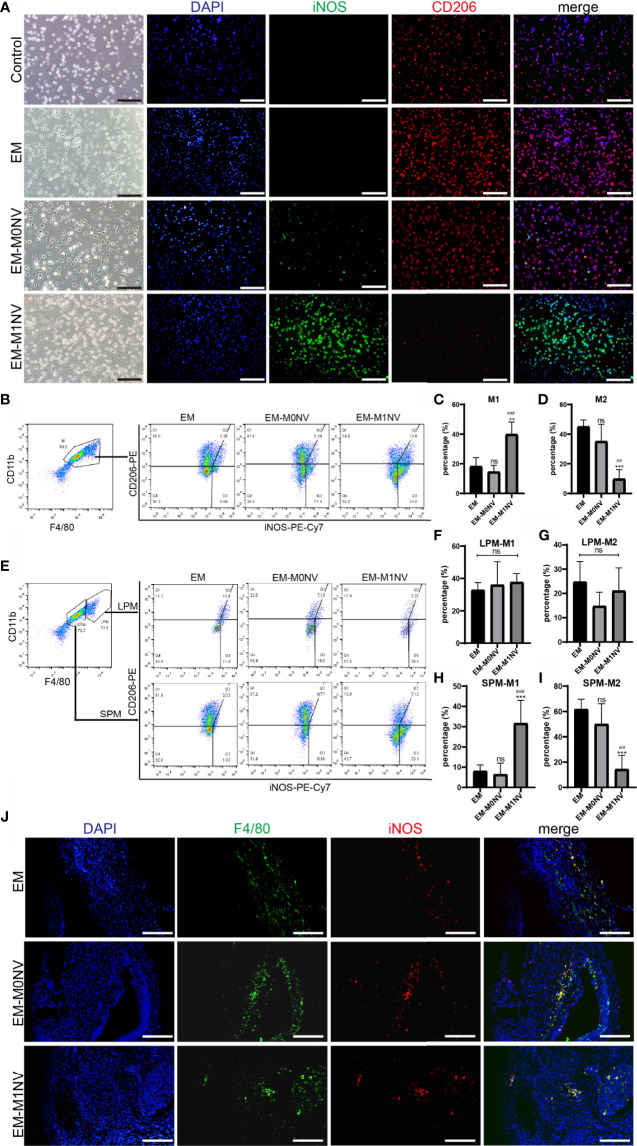
M1NVs inhibited the development of endometriosis in *vivo* through reprogramming M2. **(A)** Immunofluorescence staining of peritoneal macrophages from different groups for iNOS (green, M1 marker) and CD206 (red, M2 marker) (Scale bar = 100 μm). **(B–D)** FCM analysis of the peritoneal macrophages from different groups showed that M1NVs treatment significantly increased M1 **(C)** and reduced M2 **(D)** macrophages *in vivo*. **(E–I)** FCM analysis of the polarization of LPMs and SPMs showed that the treatment of M1NVs significantly increased M1 and reduced M2 macrophages derived from SPMs **(H, I)** while little affected the polarization of LPMs **(F, G)** (ns, No significance when compared with EM. **P<0.01, ***P<0.001 when compared with EM. ^##^P<0.01, ^###^P<0.001 when compared with EM-M0NV). **(J)** Immunofluorescence staining of macrophages in endometriosis lesions from different groups for F4/80 (green, pan marker of macrophages) and iNOS (red, M1 marker) (Scale bar = 100 μm).

To investigate whether M1NVs could program peritoneal macrophages into M1-phenotype *in vivo*, peritoneal macrophages were collected and subjected to immunofluorescence staining. The purity of macrophages was over 93% (**Supplemental Figures 1A, B**). Macrophages derived from the control group showed no expression of iNOS, a classical M1 macrophage marker, and a weak expression of CD206, a marker of M2 macrophages. Macrophages collected from the EM-M1NV group showed high expression of iNOS and low expression of CD206 compared with those collected from the EM and EM-M0NV groups (**Figure 6A**).

The exact percentages of M1 and M2 macrophages among the peritoneal macrophages were determined using FCM. M1NV treatment significantly increased the percentage of M1 macrophages and decreased that of M2 macrophages compared with those measured in the EM group treated with an equal volume of PBS, whereas M0NV treatment did not have the similar effects (**Figures 6B–D**). Our previous study showed that LPMs show different changes in polarization compared with SPMs (20). LPMs tend to polarize toward the M1 phenotype, whereas SPMs tend to polarize towards the M2 phenotype in our mouse model of induced endometriosis. To explore the target cells of M1NVs, we further analyzed the polarization of LPMs and SPMs separately. Interestingly, FCM revealed that LPMs showed similar polarization trends, regardless of the treatment (**Figures 6E–G**); the percentage of M1 macrophages was higher than that of M2 macrophages. In contrast, SPMs showed different trends depending on the treatment (**Figures 6E, H, I**). M1NV treatment distinctly increased the population of M1 macrophages and decreased that of M2 macrophages in SPMs compared with M0NV and PBS treatments, which was consistent with the overall trends in peritoneal macrophage polarization. Taken together, these findings suggested that M1NVs might function by targeting SPMs rather than LPMs.

We further investigated the macrophages in endometriosis lesions with immunofluorescence staining. There seemed to be no difference in M1 among the groups (**Figure 6J**).

### Safety of M1NV Treatment

**Figure 7:**
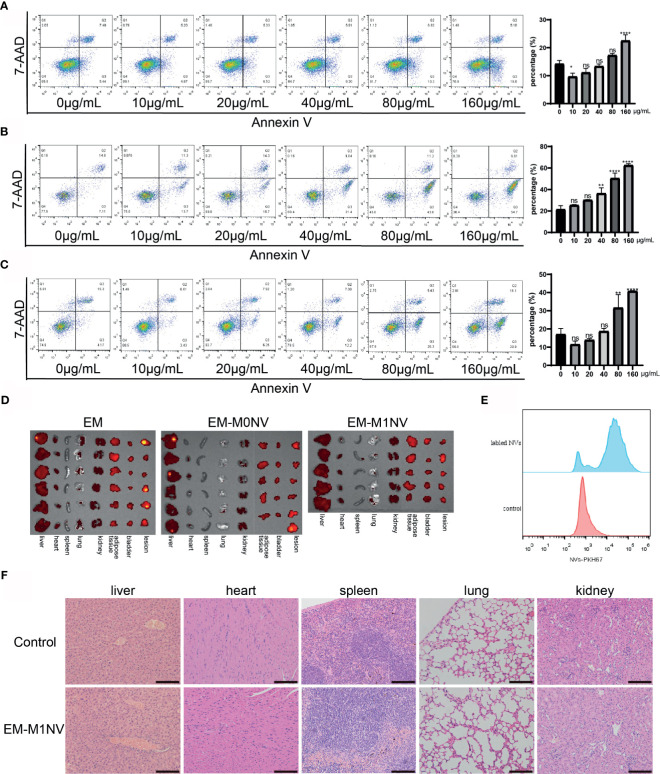
M1NVs treatment did not induce cell apoptosis and did not injury the organs. **(A)** The apoptosis percentage of macrophages treated with different concentration of M1NVs. **(B)** The apoptosis percentage of ESCs treated with different concentration of M1NVs. **(C)** The apoptosis percentage of HUVECs treated with different concentration of M1NVs. ns, No significance when compared with the group treated with 0 μg/mL M1NVs. *P<0.05, **P<0.01, ****P<0.0001 when compared with group treated with 0 μg/mL M1NVs. **(D)** IVIS of the organs from different groups. Group EM was the negative control since no labeled NVs were injected. **(E)** FCM showed NVs labeled with PKH67 were engulfed by the peritoneal macrophages. **(F)** H&E staining sections of organs from control group and EM-M1NV group (Scale bar = 100 μm).

The aforementioned results demonstrated the therapeutic efficiency of M1NVs. Therefore, we investigated the safety of M1NV treatment. RAW264.7 cells, ESCs, and HUVECs were treated with different concentrations of M1NVs, and cell apoptosis was assessed. Results showed that M1NVs did not induce apoptosis in RAW264.7 cells at concentrations lower than 160 μg/mL. M1NV inhibited apoptosis in RAW264.7 cells at a concentration of 10 μg/mL (**Figure 7A**). In ESCs, the percentage of apoptotic cells increased when the concentration of M1NVs increased to greater than 40 μg/mL, although no significant differences were observed in cell apoptosis at a concentration of 20 μg/mL, a concentration used in our *in vitro* experiments (**Figure 7B**). A similar phenomenon was observed in HUVEC cultures (**Figure 7C**). Thus, apoptosis analysis indicated that treatment with an appropriate concentration of M1NVs did not have toxic effects on cells *in vitro.* Additionally, inhibition of ESC migration and invasion and HUVEC tube formation was not a consequence of cell apoptosis.

Next, we evaluated the effects of M1NV treatment on organ injury *in vivo* using IVIS. IVIS images showed no significant differences in fluorescence intensity among the EM, EM-M0NV, and EM-M1NV groups. PKH67-labeled NVs did not accumulate in the organs, including the liver, heart, spleen, lungs, kidney, adipose tissue near the bladder, and bladder, and in lesions (**Figure 7D**), but these NVs were phagocytosed by peritoneal macrophages (**Figure 7E**), which might explain the lack of NV accumulation in the organs. H&E staining showed no structural alterations in the organs (**Figure 7F**). These results indicated that M1NV treatment was feasible and safe to use with no obvious induction of organ injury. Therefore, our findings provide a basis for further studies on treating endometriosis with engineered NVs.

## Discussion

Endometriosis is an inflammatory disease, in which the dysfunction of macrophages plays a critical role. In this study, we investigated the effects of nanovesicles derived from M1 macrophage (MINVs) on the development of endometriosis. NVs derived from macrophages obtained through serial extrusion showed characteristics similar to those of exosomes derived under natural conditions. The NVs could be internalized by ESCs, HUVECs, and macrophages. Additionally, we showed that M2-like macrophages could be reprogrammed into M1-like macrophages by treatment with M1NVs. M1NVs directly and indirectly inhibited the migration and invasion of EM-ESCs *in vitro*. Moreover, M1NVs reduced HUVEC tube formation directly or through reprogramming of M2 macrophages into M1 macrophages. The Re-M2 macrophages then blocked the ability of EM-ESCs to promote tube formation *in vitro*. The *in vivo* experiments indicated that M1NVs could inhibit the development of endometriosis, partially *via* reprogramming of M2 macrophages. Importantly, M1NVs did not cause damage to any organs in the *in vivo* experiments.

EVs have emerged as crucial mechanisms that facilitate communication between different cells, and between cells and the environment. These 30–5000 nm vesicles transport lipids, proteins, DNAs, and RNAs to recipient cells, inducing functional alterations (24). EVs can be stored for long durations, with reduced loss of function compared with cells applied in therapeutic strategies (25). Moreover, the cargo encapsulated by EVs or NVs has showed more stable functions than free cargo (26–28), suggesting the potential applications of EVs or NVs in drug delivery (29). Previous studies have shown that mechanical extrusion is a feasible method for producing NVs (30, 31); in addition, NVs derived from M1 macrophages contain specific RNAs (5). Vesicles derived from macrophages present integrins which confer upon EVs the capacity of targeting inflamed tissues (31, 32). Additionally, alterations in membrane markers by hybridization with the membrane can further enhance the targeting capacity of EVs (33) and the cargo encapsulated by EVs could be altered by engineering technology, which can enhance the functions of EVs (28, 34, 35). This study revealed that M1NVs could successfully reprogram M2-like macrophages into M1-like macrophages, consistent with a previous study (5). These features and effects of M1NVs made it to be a promising tool in the treatment of endometriosis.

Endometriosis is a benign disease with a tendency for invasive growth (36, 37). Abnormal angiogenesis in endometriosis lesions is similar to that observed in tumor growth (38, 39). Factors closely associated with ovarian cancer have been discovered in endometriosis. For example, the tumor suppressor molecule E-cadherin is not expressed in endometriosis tissues (36). Additionally, long noncoding RNA *TC0101441*, hypoxia-inducible factor-1α, and overexpressed chloride channel-3 have been shown to promote the migration and invasion of EM-ESCs (21, 38, 39). Previous studies have also shown that angiogenesis is essential for the establishment and development of endometriosis (40, 41). New vessels emerging from endometriosis lesions further guide the generation of nerve fibers and exacerbate the pain associated with endometriosis (42, 43). In this study, we demonstrated that EM-ESCs promoted angiogenesis compared with Co-ESCs.

Cell migration and invasion, and angiogenesis are important characteristics of endometriosis and are at least in part mediated by M2 macrophages (11, 44). Notably, in our study, M1NVs inhibited the migration and invasion of EM-ESCs directly or by reprogramming M2 macrophages. The same effects were observed for angiogenesis. The Re-M2 macrophages reversed the angiogenesis-promoting effects of EM-ESCs. Although the microvessel density did not significantly differ among the groups, the structures of lesions in the EM-M1NV group were disrupted (data not shown). Taken together, these results suggested that M1NVs may block the development of endometriosis through several mechanisms related to inhibition of EM-ESC migration and invasion, and angiogenesis. Although VEGFA gene expression in the macrophages was changed, we could not identify whether this was the main mechanism, since the function of M1 macrophage is very complicated, and several factors might be involved in the inhibition of endometriosis. A further inhibition and rescue experiment associated with VEGFA should be designed to investigate the role of VEGFA in the research. Although the exact mechanisms were not determined, the inhibitory effects of M1NVs suggested that M1NVs may have application in the treatment of endometriosis. Further studies are needed to investigate the molecular mechanisms of these effects and to establish novel targets for treatment.

The inhibitory effects of M1NVs on endometriosis were largely achieved by the regulation of macrophage function. Macrophages, which are critical in endometriosis, can be divided into the M1 and M2 phenotypes (4, 43). Several researches reported that M2 macrophage promoted the migration and invasion of ESCs, angiogenesis, and fibrosis in endometriosis (11–13, 15, 44). Previous studies have demonstrated the antitumor efficacy associated with switching from the M2 to the M1 phenotype (5, 45). In this study, we confirmed that M1NVs could effectively reprogram M2 macrophages into M1 macrophages both *in vitro* and *in vivo*. Moreover, switching of M2 macrophages to M1 macrophages potently inhibited the development of endometriosis.

Our *in vivo* experiments showed that M1NVs could reprogram M2 macrophages to M1 macrophages, but the effects on LPMs and SPMs differed. Our previous study showed that SPMs polarize to M2 macrophages, whereas LPMs tend to polarize to M1 macrophages (22). Consistent with these findings, Hogg et al. illustrated the protective effects of monocyte-derived LPM when the peritoneal cavity is challenged with ectopic endometrium (46). In this study, we observed that M2 macrophages were mainly differentiated from SPMs, and these macrophages were susceptible to M1NVs, whereas M2 macrophages differentiated from LPMs were not easily affected by M1NVs. Although M1NVs could reprogram M2 macrophages into M1 macrophages, they did not affect the conversion between LPMs and SPMs. Previous studies have reported the pro-inflammatory status of SPMs and the pro-repair status of LPMs (47), contradictory to our findings. These contradictory results may be attributed to progressive phenotypic changes in macrophages during the development of endometriosis (48). In the current study, SPMs were susceptible to stimulations by M1NVs, whereas LPMs tended to maintain a stable phenotype. Moreover, M1NV treatment did not alter the number of lesions, but significantly reduced the volume and weight of endometriosis lesions. We injected M1NVs on day 7 after injection of the uterus fragments, when the endometrial fragments had implanted into the peritoneum. According to studies by Johan et al., M1 macrophages are the dominant phenotype in the first week, and subsequently, M2 macrophages can increase to form the main subset (48). Therefore, day 7, after the injection of fragments, may be the appropriate time point for treating endometriosis. However, further studies are needed to determine whether M1NVs also played roles in the implantation of endometriosis lesions.

In this study, we used intraperitoneal injection of M1NVs, a contrast to previous studies (5, 28). EVs injected intravenously accumulate in the liver, lungs, and spleen due to the large amounts of macrophages in these tissues, and could potentially damage the organs. The peritoneal cavity, which is the main location of endometriosis lesions, has an abundance of macrophages. M1NVs injected into the peritoneal cavity are immediately engulfed by the macrophages, and we believe that few are absorbed into the blood. Our findings showed that M1NVs injected intraperitoneally did not accumulate in the organs; accordingly, intraperitoneal injection may be the suitable method for treating endometriosis with M1NVs.

This study had some limitations. First, although M1NVs could effectively reprogram M2 macrophages, we did not explore which molecules contained within the M1NVs might exert the above-mentioned effects. Hence, further research is needed to investigate the mechanisms through which M1NVs reprogram the macrophages. Additionally, immune responses *in vivo* are complex and involve adaptive immune responses. Some researchers have reported that EVs from activated dendritic cells can effectively induce cellular and humoral immunity (34). In addition to macrophages, T-cell subsets in the peritoneal cavity are also altered in mice with endometriosis (22). Therefore, we were unable to exclude the involvement of adaptive immune responses in M1NV-induced inhibition of endometriosis. Besides, the effect of M1NVs on LPM and SPM differed. A further study should be designed to investigate the exact effect of M1NVs on M2 derived from SPM and explore the mechanism by which M1NV works.

## Conclusion

In summary, this study provided evidence that M1NV treatment reprogrammed endometriosis-promoting M2 macrophages into endometriosis-inhibiting M1 macrophages, inhibited the migration and invasion of EM-ESCs, and blocked angiogenesis. Intraperitoneal injection of M1NVs could suppress the development of endometriosis with good safety and efficacy. Thus, our findings suggest a novel method for treating endometriosis using M1NVs.

## Data Availability Statement

The raw data supporting the conclusions of this article will be made available by the authors, without undue reservation.

## Ethics Statement

The studies involving human participants were reviewed and approved by Ethics Committee of Qilu Hospital of Shandong University. The patients/participants provided their written informed consent to participate in this study. The animal study was reviewed and approved by Animal Care and Use Committee of Qilu Hospital of Shandong University.

## Author Contributions

GW conceived and designed the study. QL performed the experiments, analyzed, interpreted data and drafted the manuscript. MY, XJ, YH, JL, DL, and MJ performed the experiments. All authors participated in the writing and revision of the manuscript and agree to be accountable for the content of the work. All authors contributed to the article and approved the submitted version.

## Funding

This work was supported by the National Natural Science Foundation of China [grant numbers 82071621, 81771552, and 81901458], the Key Technology Research and Development Program of Shandong [grant number 2019GSF108071], and the Clinical Practical New Technology Development Fund of Qilu Hospital of Shandong University [grant number 2019-23. The funders had no role in study design; the collection, analysis and interpretation of data; the writing of the report; and the decision to submit the article for publication.

## Conflict of Interest

The authors declare that the research was conducted in the absence of any commercial or financial relationships that could be construed as a potential conflict of interest.

## Supplementary Material

Supplementary Figure 1
**(A)** The adherent cells were identified with FCM. Over 93% of the cells were macrophages. “negative control” means no anti-CD11b and anti-F4/80 antibodies. **(B)** The adherent cells expressed F4/80, which is the pan marker for macrophages (Scale bar =100 μm).
