# Supplementary figures and images for: M1 Macrophage-Derived Nanovesicles Repolarize M2 Macrophages for Inhibiting the Development of Endometriosis
Source: Front Immunol. 2021 Jul 20;12:707784. doi: 10.3389/fimmu.2021.707784 (PMC8329654; doi:10.3389/fimmu.2021.707784)

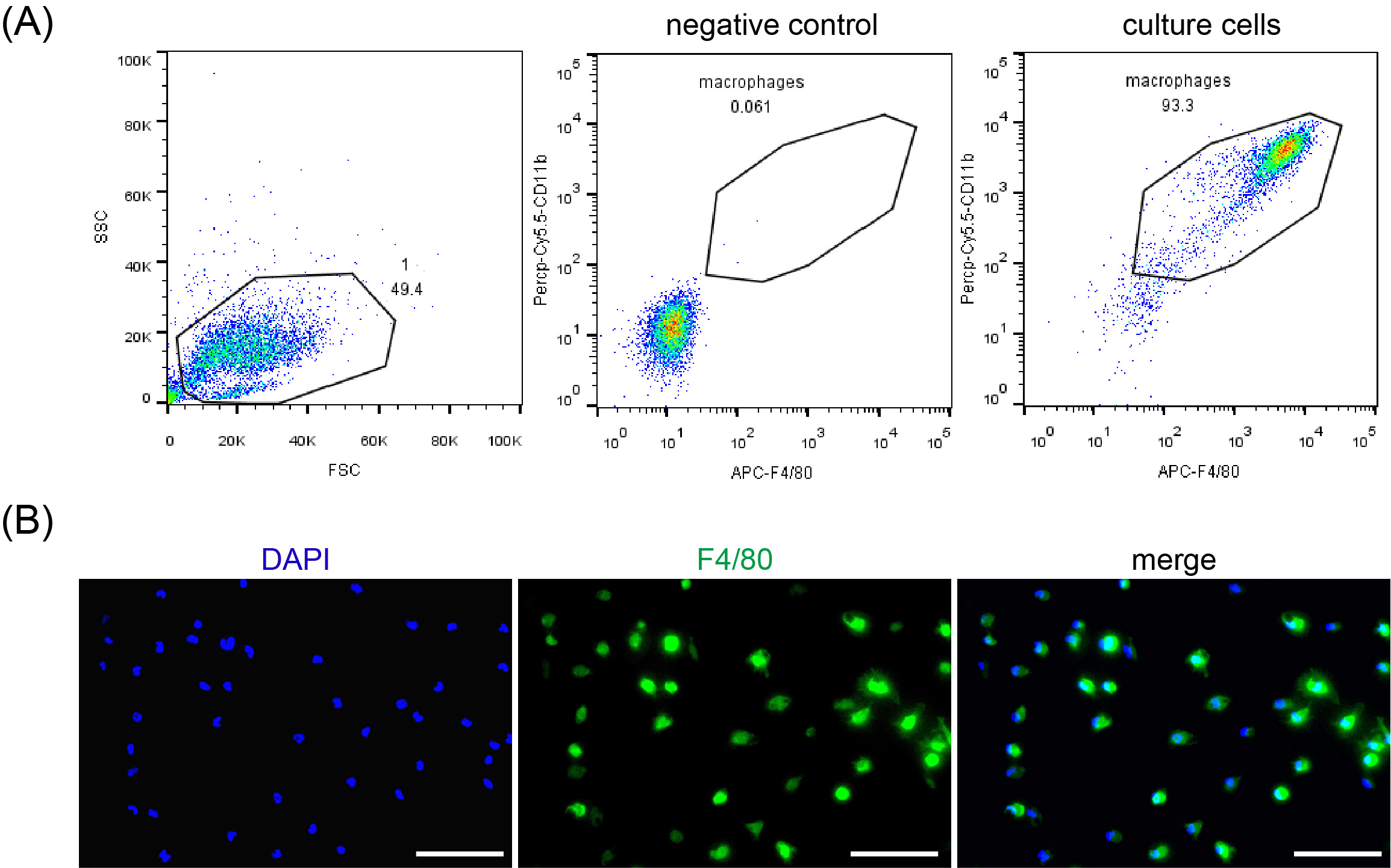

Supplement: Supplementary Figure 1 — (A) The adherent cells were identified with FCM. Over 93% of the cells were macrophages. “negative control” means no anti-CD11b and anti-F4/80 antibodies. (B) The adherent cells expressed F4/80, which is the pan marker for macrophages (Scale bar =100 μm). [file Image_1.jpeg]
